# Trends and projection of forearm fractures including elbow fractures of the Olecranon in Sweden: an analysis of 363 968 fractures using public aggregated data

**DOI:** 10.1186/s12891-023-07162-7

**Published:** 2024-01-05

**Authors:** Michael Axenhus, Simon Svedman, Martin Magnéli

**Affiliations:** 1 Department of Orthopaedic Surgery, Danderyd Hospital, Stockholm, Sweden; 2 Department of Molecular Medicine and Surgery, Karolinska Institutet, Stockholm, Sweden; 3 Department of Orthopaedic Surgery, Karolinska University Hospital, Stockholm, Sweden; 4 Department of Clinical Sciences at Danderyd Hospital, Karolinska Institutet, Stockholm, Sweden

**Keywords:** Forearm fractures, Trends, Regional variations, Sex differences, Preventive measures

## Abstract

**Background:**

Forearm and olecranon fractures are a common orthopaedic injury. This study aimed to analyse whether the incidence of forearm injury is changing and identifying trends in the number of forearm and olecranon fractures using public aggregated data in Sweden.

**Methods:**

The number of forearm and olecranon fractures as defined by the number of registered diagnoses with the ICD-10 code of S52 were collected and normalized per 100,000 inhabitants and stratified per sex, age, and month. Age-adjusted incidence for forearm and olecranon fractures were calculated using the direct method. Poisson regression was used to analyse monthly, seasonal and yearly change in forearm and olecranon fracture incidence. Logistical regression was used to predict future trends of forearm and olecranon fractures.

**Results:**

The findings revealed a slight decreasing trend in forearm and olecranon fractures. The average incidence rate during the study period was 333 with women having a higher incidence rate than men. More fractures occurred in the winter months. Fluctuations in the number of forearm and olecranon fractures were observed during 2020 which may be influenced by the COVID-19 pandemic. Based on current data, forearm and olecranon fractures are expected to decrease in Sweden by 2035.

**Conclusion:**

This study describes the trend of forearm and olecranon fractures among individuals according to sex and age in Sweden using easily obtainable data. Trends in forearm and olecranon fractures are dependent on sex and age but generally show a decreasing trend. More precise studies are needed in order to properly quantify the specific incidence of various subtypes of forearm and olecranon fractures and associated risk factors.

## Introduction

Forearm fractures including distal radial fractures (DRF) and fractures of the olecranon are some of the most common orthopaedic injuries. Fractures affecting the hands and upper extremities can have profound impacts on the well-being and independence of individuals [1, 2]. Understanding the trends and patterns of forearm and olecranon fractures (FOF) is therefore crucial for developing effective preventive measures, allocating healthcare resources, and improving the quality of care.

FOF are almost always caused by trauma, and is influenced by various factors such as decreased bone density, muscle weakness, balance problems, and age-related physiological changes [3]. These fractures not only lead to physical pain and functional limitations but also increase the risk of hospitalization, disability, and mortality [2, 4]. Although FOR can be successfully treated with either surgery or casts [5], efforts should be made to decrease the incidence of fractures as they impose a substantial economic burden on healthcare systems due to the costs associated with hospital admissions, surgical interventions, rehabilitation, and long-term care [6, 7].

Sweden, like many other countries, is experiencing the consequences of population aging. As such, it is essential to monitor and analyse the trends in FOF among the population to identify high-risk groups, target preventive interventions, and allocate healthcare resources effectively.

This study aims to analyse the trends in the number of FOF in Sweden, with a specific focus on sex, age and temporal differences. By examining these trends and patterns, we can gain valuable insights into the epidemiology of FOF and identify areas for intervention and prevention or groups at risk.

## Methods

### Study design and data source

This retrospective population-based study utilized data from the Swedish National Board of Health and Welfare (SNBHW). The SNBHW contains information on hospital admissions, outpatient specialist visits, and diagnoses for all individuals treated in Swedish hospitals. The SNBHW diagnosis register provides data on all diagnoses in Sweden, including cause of death in the national patient register (NPR) [8]. These national registers ensure comprehensive coverage of healthcare utilization and mortality data, allowing for a robust analysis of fractures amongst the population [9]. The NPR provides public aggregated data on a population basis. ICD-10 codes are reported per unique personal identification number and is counted only once per year and diagnosis group, minimizing the risk of double reporting.

### Study population

The study population comprised individuals residing in Sweden that experienced a forearm or an olecranon fracture during the time periods of 2015–2021 and 2013–2019. We included data from two seven-year period, specifically from January 1st, 2015, to December 31st, 2021, and January 1st, 2013, to December 31st, 2019. We chose two different time periods in order to account for the COVID-19 pandemic and its influence on diagnostics. The timeframe chosen allowed for a comprehensive analysis of temporal trends in FOF with a long enough time span for future trend projection.

### Identification of forearm fractures

FOF were identified based on the International Classification of Diseases, Tenth Revision (ICD-10) codes. Specifically, we include all fractures of the radius, ulna and olecranon (ICD-10 codes S52) and extracted the relevant cases from the NPR diagnoses register, a total of 363 968 cases during the study period.

### Data analysis

First, we calculated the overall annual incidence of FOF per 100,000 person-years and stratified this data according to sex and age groups. Data were categorized primarily by sex, and age of under and over 65 years of age. We chose 65 years of age as a cut-off point due to the higher incidence of fractures in this population because of lower bone density. We did not include patients younger than 18 years old. The incidence rates were calculated by dividing the number of FOF by the age-specific population rates where the weights are taken from the population distribution of a standard population estimates obtained from Statistics Sweden [10]. Mean incidence was calculated from the number of fractures and the study population.

To assess the temporal trends in FOF, we plotted the annual incidence rates over the study period. We used Poisson regression as a trend predictor for FOF. Actual values were compared to predicted values and any significant difference is described. Age stratified data was further stratified into months in order to detect seasonal variances in the incidence of FOF amongst various age groups. A *P*-value of < 0.05 was considered statistically significant.

Furthermore, we conducted stratified analyses by sex to explore potential differences in forearm and olecranon fracture rates. Sex-specific incidence rates were calculated, and linear regression analysis was performed for each group. All calculations were performed using SPSS (Version 25.1).

### Ethical considerations

The data used in this study was publicly available, anonymized, de-identified and thus not subject to ethical review.

## Results

### Fracture incidence

**Fig. 1:**
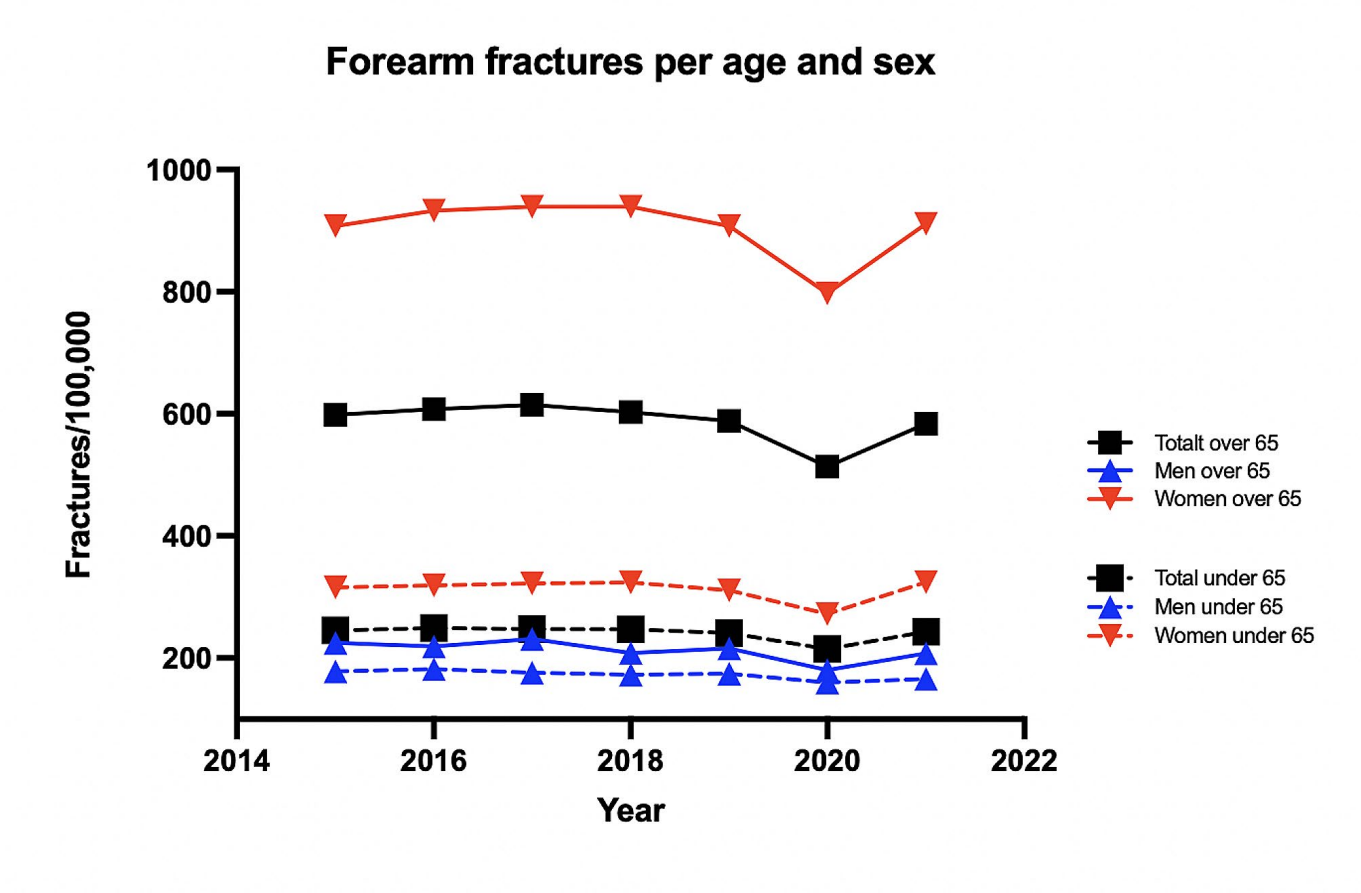
Age and sex distribution of forearm and olecranon fracture incidence rates in Sweden 2015–2021

Between 2015 and 2021 there were an average of 25 867 (range 23 312–26 722) patients registered with a FOF per year in Sweden. From 2015 to 2019 there was no significant change in any group studied. There was a significant drop in incidence during 2020 with a rebound of fractures during 2021 (Fig. 1). The reduction in fracture incidence during 2020 was significant for men (*p* < 0.001) and women over 65 (*p* < 0.001) years of age compared to expected outcome (Table 1).

**Table 1:** Fracture number and fracture incidence rates amongst men and women during 2015–2021

Year	All patients		Men		Women	
	Incidence rate (/100,000)	No. Patients with fractures	Incidence rate (/100,000)	No. Patients with fractures	Incidence rate (/100,000)	No. Patients with fractures
2015	335	25 462	187	7071	480	18 391
2016	340	26 224	189	7233	490	18 991
2017	341	26 534	187	7247	494	19 287
2018	339	26 588	180	7045	496	19 543
2019	331	26 227	183	7246	478	18 981
2020	293	23 312	164	6522	421	16 790
2021	333	26 725	175	7026	491	19 699

### Age and sex distribution

**Fig. 2:**
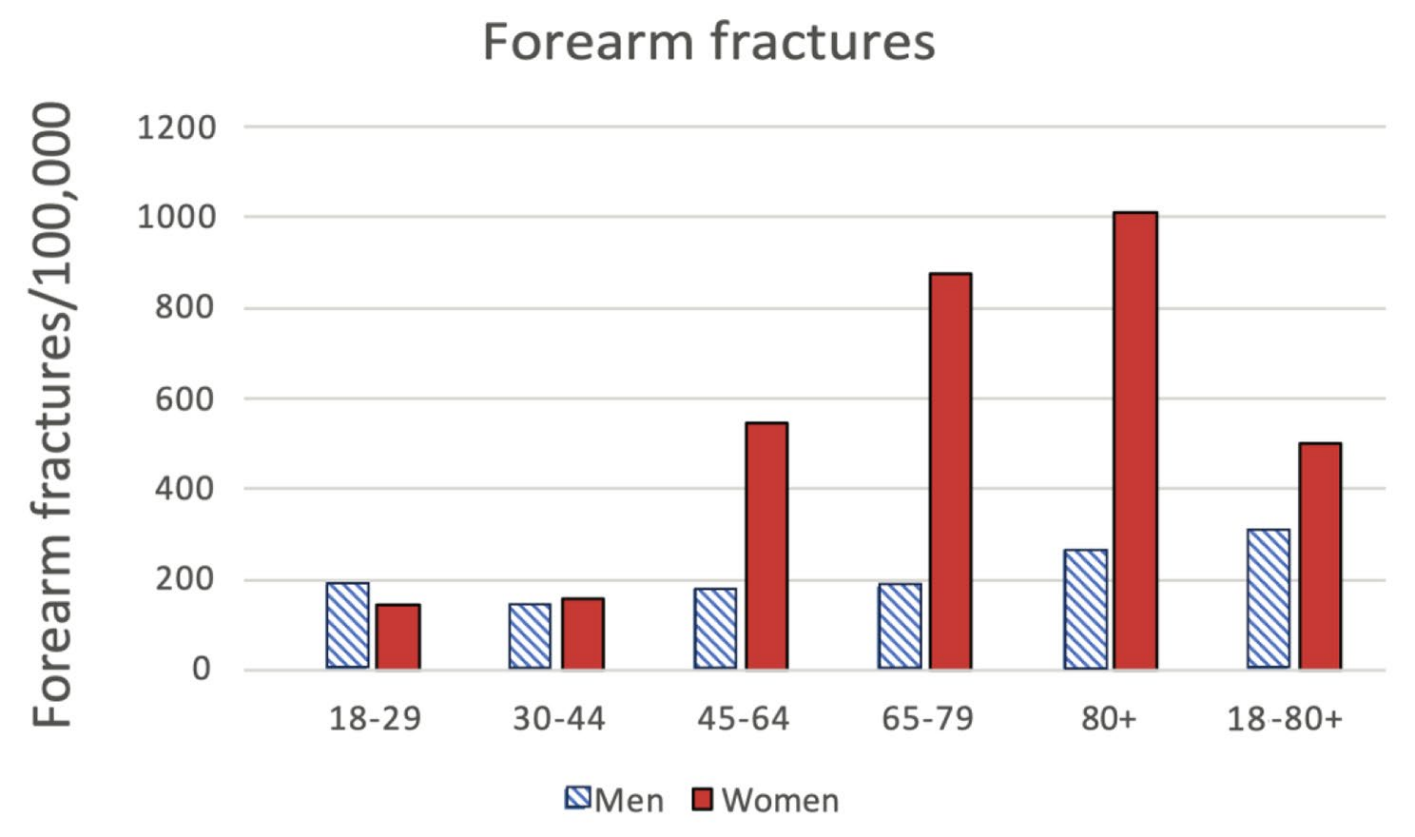
Age distribution for forearm and olecranon fractures in Sweden during 2021

Age- and sex-specific incidence rates per 100,000 person-years of FOF were calculated (Fig. 2). In women, the incidence of fracture increased steadily from the fifth decade to reach a peak of 1005/100,000 per inhabitants in the 80 + age group. In men, incidence remained low until the eighth decade, from which point it rose to a peak of 265/100,000 per inhabitants in the 80 + age group.

### Seasonal variance in fracture incidence

**Fig. 3:**
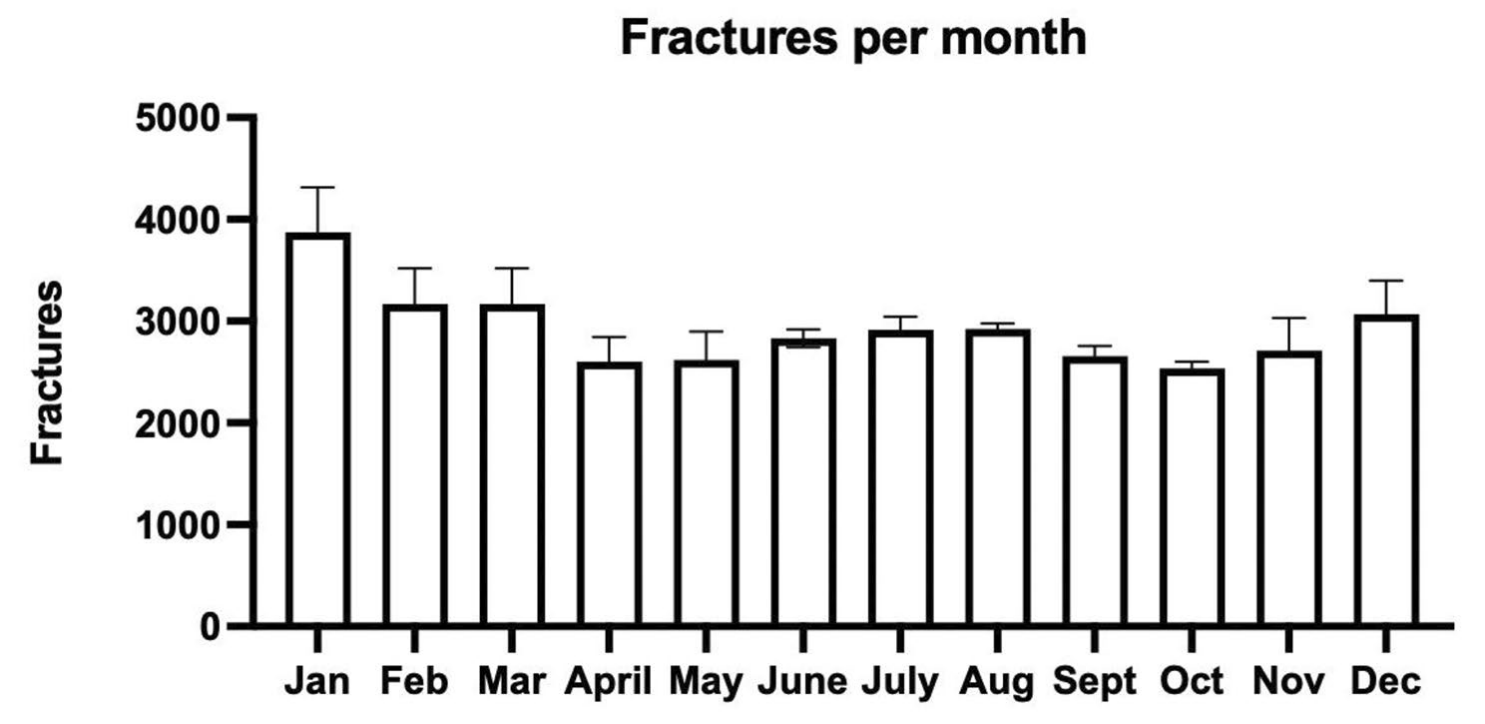
Monthly distribution of forearm and olecranon fractures during 2015–2021

Cumulative data demonstrated a statistically significant increase in fracture incidence during the winter months (December-February) (Fig. 3).

**Fig. 4:**
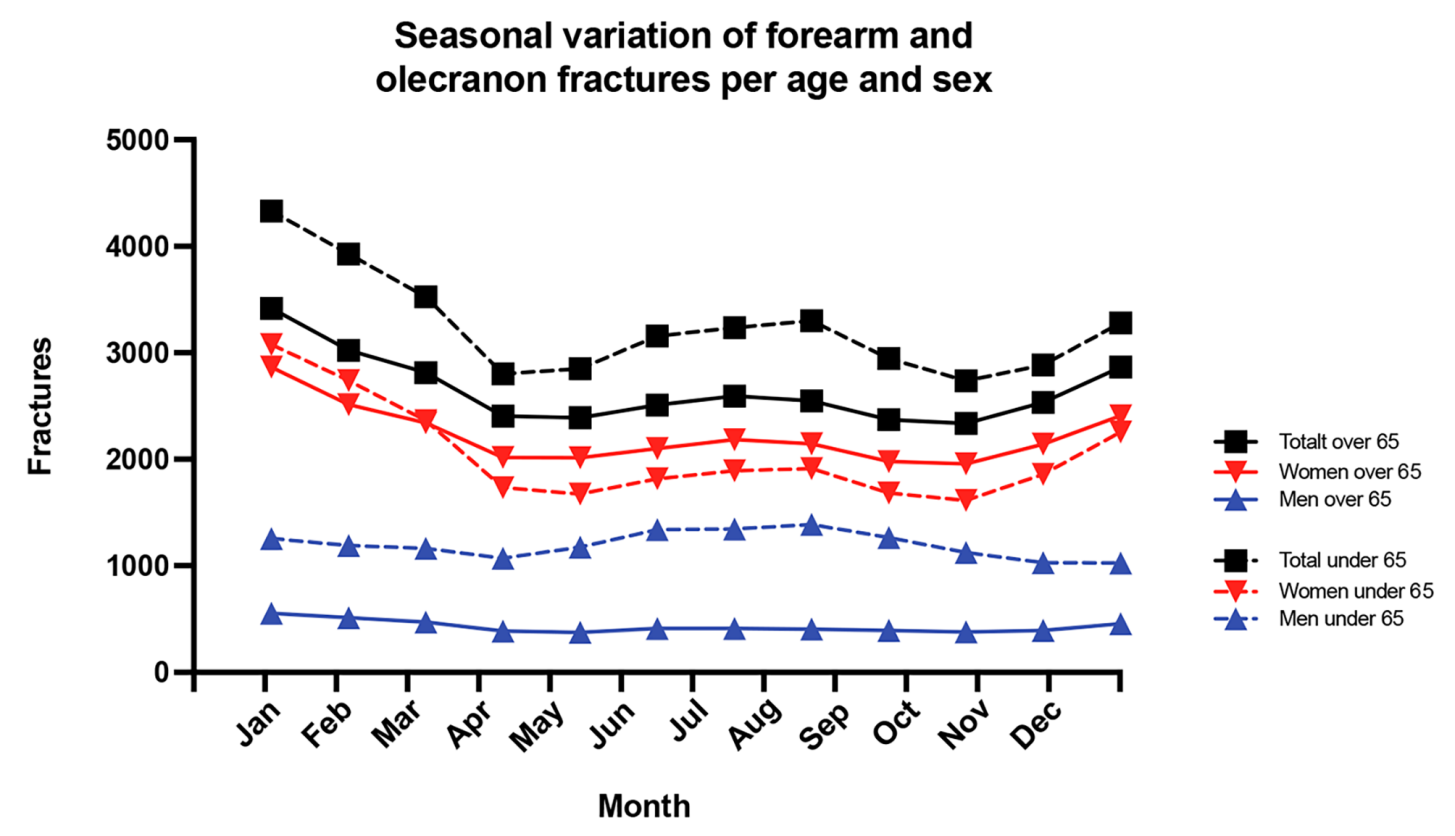
Seasonal variance in forearm and olecranon fractures per age and sex. Average mean during 2015–2021

Fracture incidence rate tended to decrease during autumn and early spring. Across the seven-year period, 28% of fractures occurred in winter compared with 24%, 25% and 23% in spring, summer and autumn, respectively. Women and men over 65 years of age had a higher fracture incidence rate compared to women and men aged 64 years or younger (Fig. 4).

### Fracture projection analysis

**Fig. 5:**
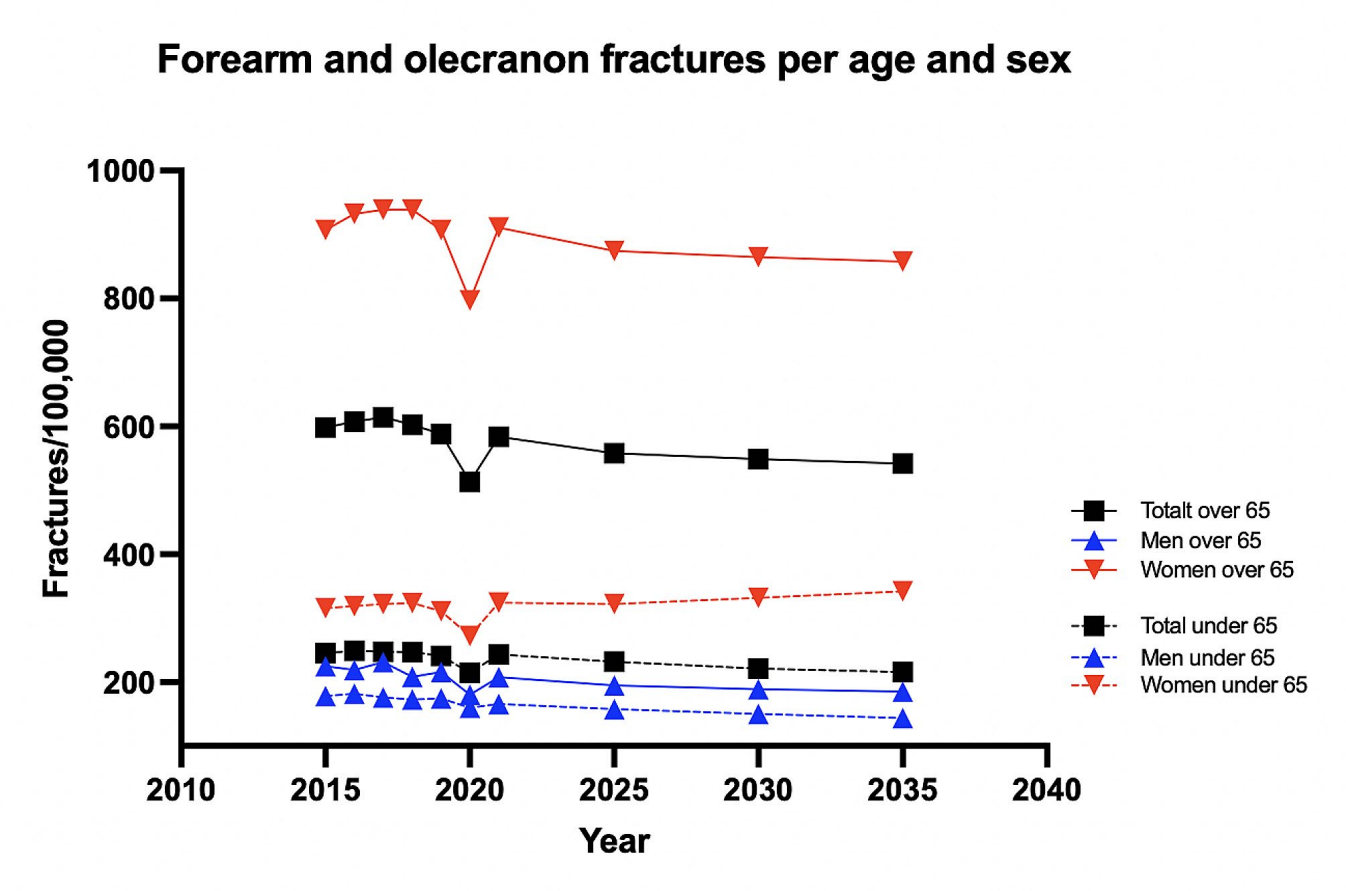
Projection of the incidence of FOF in men and women

A stable but slowly decreasing trend of FOF was demonstrated during the next 15 years (Fig. 5). Assuming that the identified trend continues, by 2035 there will be a 12% decrease of FOF amongst men and women aged 65 or older and a decrease of 7% amongst men and women aged 64 or younger compared to current incidence rates (Table 2).

**Table 2:** Projected change in forearm and olecranon fracture incidence amongst men and women until 2035. 2021 value is displayed as fractures per 100,000. 2025–2035 shows expected change in incidence compared to 2021

	Men over 65	Men under 65	Women over 65	Women under 65	Totalt over 65	Total under 65
Year						
2021	208	166	911	324	584	243
2025	-6%	-5%	-4%	1	-4%	-5%
2030	-9%	-9%	-5%	4	-6%	-8%
2035	-11	-13%	-6%	7%	-7%	-12%

**Fig. 6:**
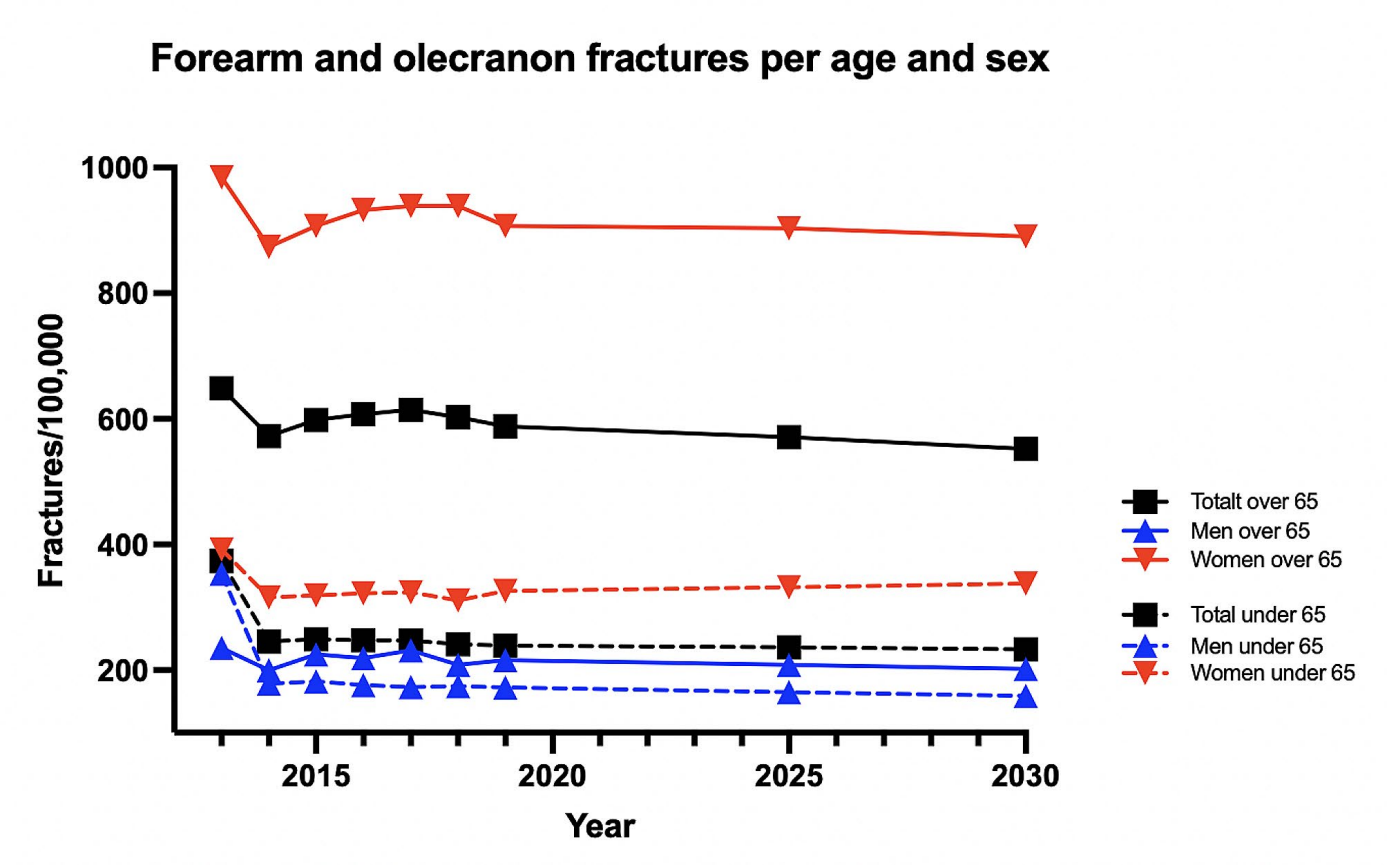
Projection of the incidence of forearm and olecranon fractures in men and women per age group excluding the pandemic years of 2020–2021

To account for pandemic influence, we also analyzed the incidence of FOF starting from 2013 but excluded the years 2020–2021 in the projection analysis. The downward trend of FOF was retained for 2025–2030 (Fig. 6). Discounting the influence of the COVID-19 pandemic, by 2030 there will be a decrease in FOF of 11% amongst people aged 65 or older and 6% amongst people younger than 65 years of age.

## Discussion

The observed stable yet high trend in FOF incidence rates among the Swedish population highlights the need for targeted preventive measures and interventions. Two groups stand out for risk of FOF, men older than 80 years and women 65 years and older. These results were not surprising given that forearm fractures are a common injury in elderly women due to osteoporosis [11]. Future trend prediction indicates lower incidence of FOF during the next 15 years, especially in people over 65 years of age and in older men. This projection was not significantly influenced by the COVID-19 pandemic.

The present study benefits from utilizing data sourced from the NPR, which encompasses all registered citizens. This reduces the potential for poor data completeness, a common issue observed in another validated Swedish register, the ‘Svenska Frakturregistret’ [12]. Previous research has mostly focused on DRF due to its importance for future hand- and wrist function. A previous study using the NPR from 2010 on DRF demonstrate an overall incidence rate of 320 per 100,000 person-years, a consistent trend since 2005 [13]. Whether the patient characteristics or mechanism of injury of non-DRF injuries, such as olecranon fractures, differ from DRF needs further examination and was not possible to analyze with the available dataset.

The finding that there were significant reductions in both incidence rates and number of FOF during 2020 has previously been reported in numerous publications from different countries [14–17]. However, to our knowledge this is the first nationwide study reporting a significant reduction in FOF during the pandemic. Sweden was famously not one of the countries subjected to harsh lockdown laws, but efforts were made to limit larger groups from partaking in indoor sports. This, together with a change in activity levels, might explain the observed decrease in FOF.

Studies from Europe, North America and Asia show how the incidence of FOF increases over time [18–20]. However, this notion has been brought into question during the last years. In similarity to our results, newer studies have shown that the change in FOF is non-existent or even decreasing in some instances [21–23] contrasted by a Swedish regional study during overlapping years, showing an increase in DRF [24].

We found that women experienced more FOF than men, and when looking at incidence in 2015 to 2021 there was a decrease in FOF among people aged 65 and older with a decreased in FOF incidence in 2021 compared to 2015, with a projected further decrease of 7% from 2021. FOF amongst people 65 years or younger is also expected to decrease in total by 12% by 2035 compared to 2021 levels. The demonstrated decrease in incidence and projection of a continuous decrease in all forearm injuries are novel and welcome and might be due to increased awareness in the society and protective gear during leisure activities in the young. We also should consider the effects of preventive strategies such as better treatment and screening of osteoporosis and increased neuromuscular activation in late adulthood.

In Sweden’s four distinct seasons, women over 65 showed increased incidence of FOF in winter, while men under 65 had fractures in both winter and summer, probably due to higher year-round activity levels. It has been described that summer activities raise fracture risks in younger individuals while winter fractures among the elderly relate to cold weather and falls [25–29]. Our study, covering a wider range of forearm injuries and ages, are in line with prior research focused on adults above 18 and specific forearm fractures as reported by Rundgren et al. in 2020 [30].

This study has some limitations. First, this study relies on publicly aggregated data, which imposes certain restrictions. For example, we were unable to distinguish between subgroups within the ICD code of S52 such as distal radius fractures and indivudal patients. Publicly aggregated data may also be subject to under-/overreporting or coding inaccuracies. Although, efforts have been made to improve the quality of data by the SNBHW through regular audits and validations. Second, this study lacks detailed clinical information since individual patients cannot be identified. Factors such as the mechanism of injury, comorbidities, osteoporosis, physical activity levels, and socioeconomic factors were therefore not available in the dataset, limiting our ability to explore potential risk factors or understand the context of FOF among the population.

Despite these limitations, this study provides valuable insights into the temporal trends, sex differences, and seasonal variations in FOF, using public aggregated data, amongst the population in Sweden.

## Conclusion

This study describes the trend of FOF among individuals according to sex and age in Sweden using easily obtainable data. Trends in FOF are dependent on sex and age but generally show a decreasing trend. More precise studies are needed in order to properly quantify the specific incidence of various subtypes of FOF and associated risk factors.

## Data Availability

The raw data sets can be obtained from the NPR directly (https://www.socialstyrelsen.se/en/statistics-and-data/statistics/statistical-databases/). The data is also available via FigShare (DOI:10.6084/m9.figshare.24658830).

## Abbreviations

DRF: Distal radius fractures
FOF: Forearm and olecranon fractures
ICD-10: International Classification of Disease Tenth Revision
NPR: National Patient Register
SNBHW: Swedish National Board of Health and Welfare
